# A scenario for heart failure during the filling phase

**DOI:** 10.1038/s41598-024-74155-4

**Published:** 2024-10-01

**Authors:** Gianni Pedrizzetti, Ryusuke Numata, Dario Collia, Giulia Pedrizzetti, Luigino Zovatto, Anirban Banerjee

**Affiliations:** 1 Department of Engineering and Architecture, University of Trieste, Trieste, Italy; 2 Division of Cardiology, Department of Pediatrics, The Children’s Hospital of Philadelphia, University of Pennsylvania, Philadelphia, PA USA; 3 Department of Cardiovascular Surgery, Icahn School of Medicine at Mount Sinai, New York, NY USA; 4 Department of Chemical Engineering, Materials, Environment, INSTM Reference Laboratory for Engineering and Surface Treatments, Sapienza University of Rome, Rome, Italy

**Keywords:** Cardiology, Biomedical engineering

## Abstract

Heart failure (HF) is a life-threating cardiac disease that develops progressively for the reduced ability of the left ventricle (LV) to pump blood into the circulation during systole. HF can also develop in patients with a preserved systolic function, typically in presence of hypertrophic cardiomyopathy (HCM). This type of HF is sometimes termed as diastolic HF, but its biomechanical origin is still unclear. This study employs a physics-based analysis of both the LV and left atrium (LA) in selected HCM patients and matched healthy subjects using 3D echocardiography and demonstrates that alteration on the LV side (stiffening) reduces the elastic recovery of the LA. Moreover, the analysis of the forces exchanged between the two chambers demonstrates that they result unbalanced, keeping the LA in a sustained stretched condition that leads to dilation. This scenario clarifies the diastolic root of the dysfunction that may likely be the cause of the spiraling of events progressing toward failure of both LA emptying and LV filling. This deeply interdisciplinary study provides a physics-based basis for both physics/engineering modeling of heart function and to cardiologists for the design of clinical studies.

**Supplementary Information:**

The online version contains supplementary material available at 10.1038/s41598-024-74155-4.

## Introduction

Heart failure (HF) is a life-threating cardiac disease that reflects the inability of the heart to sustain blood in motion. HF develops progressively: starting with small alterations that deteriorate over time and presents itself as a primary disease or as a consequence of almost all cardiac dysfunctions. The progression toward HF corresponds to modification of the cardiac geometry (shape change, dilatation, stiffening) that is known as adverse remodeling^1^.

In the most common cases HF involves the left side of the heart, as a consequence of the reduced ability of the left ventricle (LV) to contract and pump blood into the primary circulation. When the muscular tissue (myocardium) reduces its contraction, the LV begins to compensate by increasing its volume to be able to pump a sufficient amount of blood in the background of reduced contraction. The stretched muscle further impairs contraction, and compensation further dilates the ventricle spiraling into a sequence of events that lead to failure.

The pumping ability of the LV is commonly measured by the ejection fraction ($\:\text{E}\text{F}$) defined as the relative volumetric reduction from the maximum size at beginning of contraction (end-diastole, ED, being diastole the LV filling phase) to its minimum size at the end of contraction (end-systole, ES)

1 $$\:\text{E}\text{F}=\begin{array}{c}\frac{{\text{V}}_{\text{E}\text{D}}-{\text{V}}_{\text{E}\text{S}}}{{\text{V}}_{\text{E}\text{D}}}\end{array},$$

where $\:{\text{V}}_{\text{E}\text{D}}$ and $\:{\text{V}}_{\text{E}\text{S}}$ are the LV volume at end-diastole and end-systole, respectively. The previously described scenario is characterized by a reduction of the $\:\text{E}\text{F}$ (reduction of the numerator and increase of the denominator in (1)), which underlines the inability of pumping blood during systole. Therefore, it takes the name of either “systolic HF” or “HF with reduced $\:\text{EF}^{\prime\prime}$ (HFrEF).

On the other extreme, there is a scenario where HF develops without any reduction of $\:\text{E}\text{F}$, typically in presence of hypertrophic cardiomyopathy (HCM). In this scenario the LV maintains approximately its size, the tissue becomes stiffer and reduces contractile ability. The myocardium compensates by increasing its thickness to increase pumping, which makes it stiffer and so on. This progression maintains the $\:\text{E}\text{F}$, at least for significant phase, and it is characterized by myocardial hypertrophy (which can be small and not immediately recognized) and by alterations of clinical parameters during diastole, since it is more difficult to fill a stiff ventricle.

This kind of HF takes the name of either “diastolic HF” or “HF with preserved $\text{EF}^{\prime\prime}$(HFpEF)^2^.

The definition of diastolic HF originates from the fact that systolic function (clinically defined by the value of $\:\text{E}\text{F}$) is preserved while some clinical parameters of diastole are altered. However, such alterations occur in systolic HF as well and, despite the advances in the identification on the genetic origin of HCM^3,4^, a firm understanding of the biomechanical phenomena associated with the disease and with its progression toward HF is missing.

Indeed, the mechanisms that lay at the base of growth and remodeling of myocardial muscle involve a number of biological, mechanical and geometrical factors that are only partially understood^5^. The availability of models capable of reproducing the specific combination of such mechanisms leading to HF, and particularly to HFpEF, would be of invaluable relevance for cardiovascular research. They require advancements in experiments^6,7^ in computations^8–10^, and in the development of advanced analysis of clinical data^11–13^ that represent the ultimate experimental comparison and where this study aims to contribute.

In the case of HFpEF, it is important to remark that diastole is a peculiar period of the cardiac cycle because the mitral valve connecting the LV and the left atrium (LA) is open and the two chambers are connected in a single volume. During diastole blood flows from the LA to the LV through the generation of a proper sequence of forces exchanged between the tissue surrounding cardiac chambers and blood contained therein^14^. The intensity and timing of such forces can have repercussions on the adaptation of one or the other chamber^15^; more likely the LA, that is more vulnerable to small alterations because it is weaker than the LV and has a thinner muscular layer^16^. In this respect, despite clinical evidence is growing, data and models of LA remodeling are still at an early phase of development^17–19^.

This study presents an objective physics-based analysis of systole and diastole in a group of young subjects at the early stage of hypertrophy and no comorbidities. The analysis is performed on both the LV and the LA with the aim of identifying the phenomena associated with this disease and the causes that influence its progressive worsening.

## Results

The main characteristics of the enrolled population are reported in Table 1 including volumetric values. The HCM group showed that posterior and septal wall were thicker than those of the normal subjects. In addition, figures depicted that the HCM patients do not present LV volumetric alteration in both the ED and ES phases and show a small increase in LV $\:\text{E}\text{F}$, in agreement with literature. Notably, there was a significant increase in LA volumes associated with a reduction in LA $\:\text{E}\text{F}$. This is a common type of adverse LA remodeling^20^, resulting from increase of LV myocardial stiffness, which makes it difficult to empty the atrium. Therefore, the atrium, that receives blood continuously from the pulmonary veins, is subjected to a volumetric overload and to the stretching of its muscular fibers.

**Table 1:** Main clinical characteristics of the enrolled subjects and corresponding volumetric values for the left ventricle (LV) and the left atrium (LA).

	Normal (*n* = 50)	HCM (*n* = 81)	*p*
Age (years)	12 ± 3	12 ± 5	NS
Gender	52% Male	69% Male	NS
LV septal wall width (cm)	0.71 ± 0.12	1.67 ± 0.76	< 10^−6^
LV posterior wall width (cm)	0.70 ± 0.12	1.25 ± 1.49	0.01
LV $\:{\text{V}}_{\text{E}\text{D}}$ (cm^3^)	86 ± 26	93 ± 47	NS
LV $\:{\text{V}}_{\text{E}\text{S}}$ (cm^3^)	35 ± 11	37 ± 22	NS
LV $\:\text{E}\text{F}$ (%)	59 ± 3	62 ± 6	8 10^−4^
LA $\:{\text{V}}_{\text{E}\text{D}}$ (cm^3^)	36 ± 13	59 ± 32	< 10^−6^
LA $\:{\text{V}}_{\text{E}\text{S}}$ (cm^3^)	12 ± 5	26 ± 18	< 10^−6^
LA $\:\text{E}\text{F}$ (%)	67 ± 6	58 ± 9	< 10^−6^

Values are reported as average ± standard deviation, for the normal subjects (normal) and for the patients (HCM), last column reports the p-value of a t-student test for the hypothesis that the two groups are independent samples with equal mean (NS: non significant, *p* > 0.05).

**Fig. 1:**
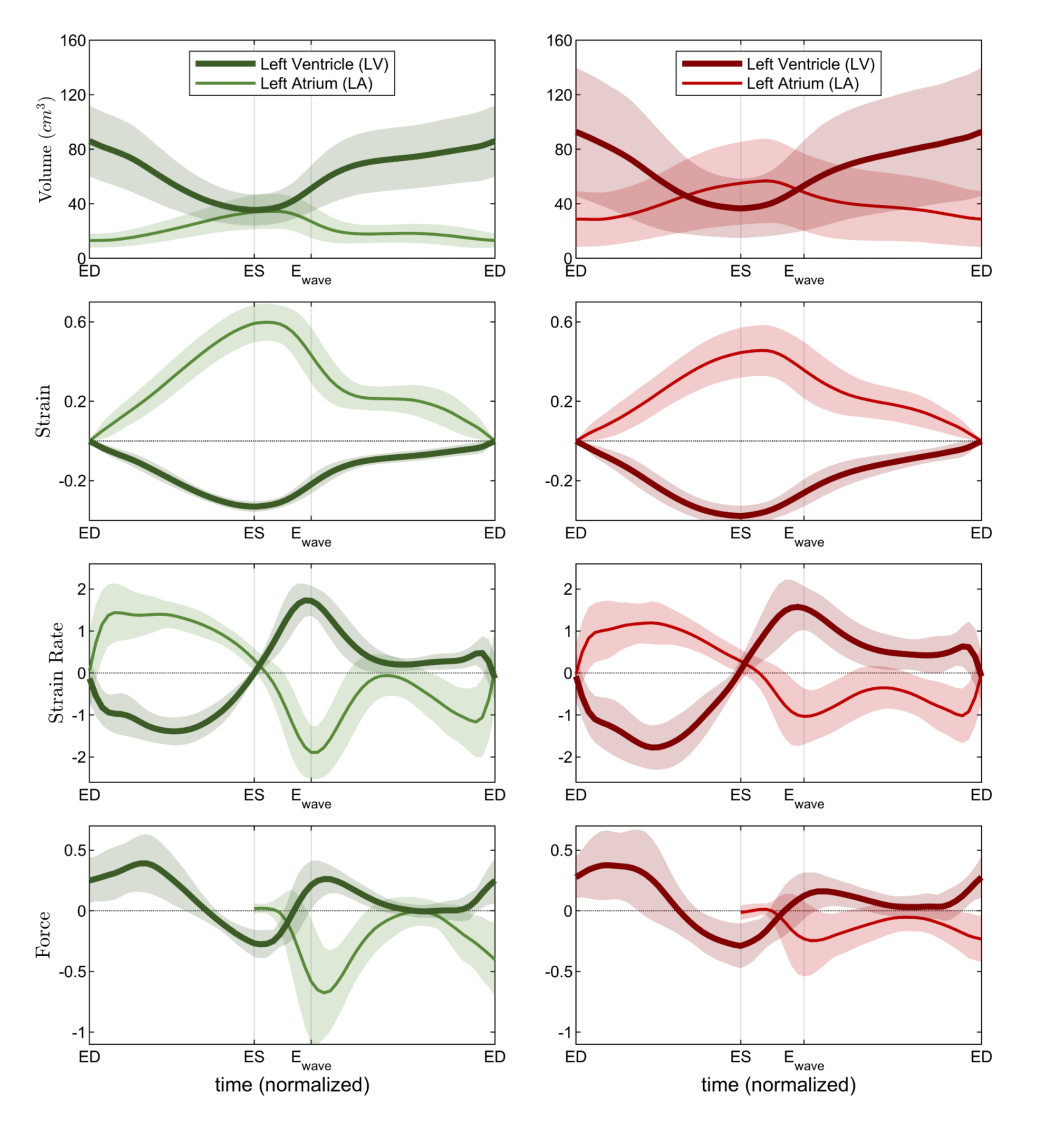
Time course of the volume, strain, strain rate, and force in the left ventricle (thick line) and in the left atrium (light line), the line reports the average value and the surrounding shadow is ± the standard deviation. Left column is evaluated from the normal population (50 subjects), right column from the matched patient with hypertrophic cardiomyopathy (81 subjects). Timing indicators are: *ED*  end diastole, *ES * end systole, *E*_*wave*_ first filling wave.

**Fig. 2:**
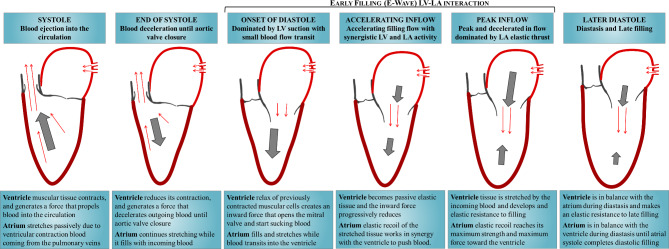
Qualitatively sketch describing the principal function phases of the normal left heart during the heartbeat.

A more comprehensive description of the mechanical function of the left heart can be extracted from the results of Fig. 1. They report, in addition to the volume, the time course of the principal strain, strain rate, and force exchanged between blood and the individual chambers. First, focus is drawn on the function in the normal subjects reported on the left column of Fig. 1, to build a reference picture. LV myocardial contraction develops during systole and the strain reaches its minimum value at end-systole, this value is considered a measure of systolic function; however, given the periodicity of the heartbeat, it also measures the amount of relaxation during diastole. When contraction is completed, the LV muscular cells relax and develop a microscopic expansion that drops LV pressure; at this point the low LV pressure actively sucks blood, opens the mitral valve and starts up diastole (LV suction phase). Afterward, the LV tissue becomes passive, it stretches during filling and progressively opposes an increasing elastic resistance to filling (inflow deceleration phase) until LV and LA reach a dynamic equilibrium. Looking at the same process from the atrial side: LA expands during systole, it is stretched by the contraction of the LV (consider that both LV apex and LA roof are approximately static, fixed at the pericardium) and by blood that is arriving from the pulmonary veins at an approximately constant rate; after diastole has begun, and the mitral valve has opened, the elastically stretched LA develops a downward force that overcomes LV elastic resistance until equilibrium is reached. This process is described visually by the sketch in Fig. 2 where particular emphasis is placed on the phases during the early phases of diastole.

**Fig. 3:**
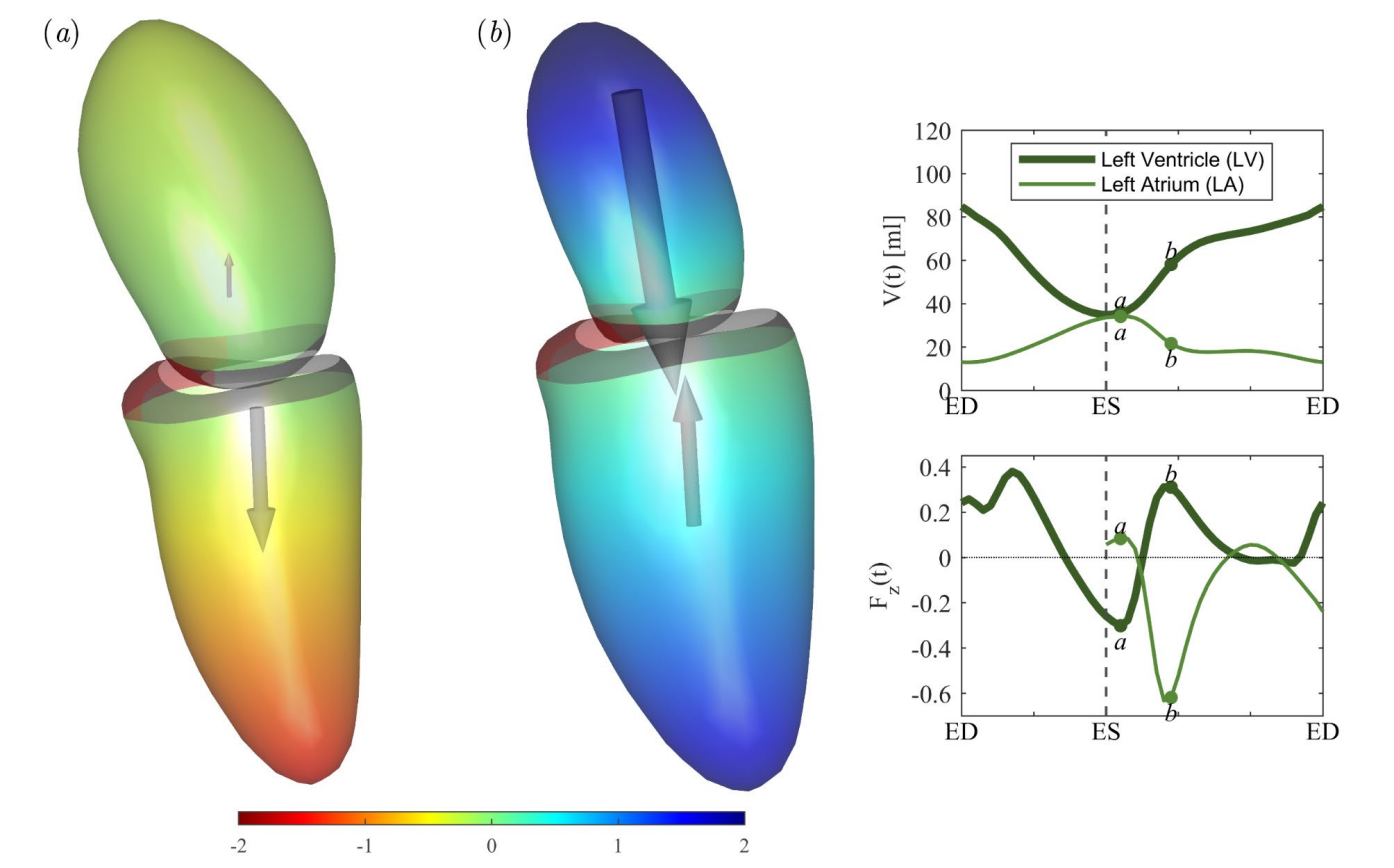
Normal heart: Overview of the force at (**a**) the beginning of diastole, or suction phase, and (**b**) before the end of early filling, or inflow deceleration phase, for an average normal subject. Pictures (**a,b**) display the geometry of left ventricle (LV) and left atrium (LA), colored by the relative pressure assumed zero at the LA-LV boundary, and the dimensionless force vector. The graphs on the right side report the time course of the volume of LA and LV (above) and the dimensionless force (below), with indication of the timing of pictures (**a,b**).

**Fig. 4:**
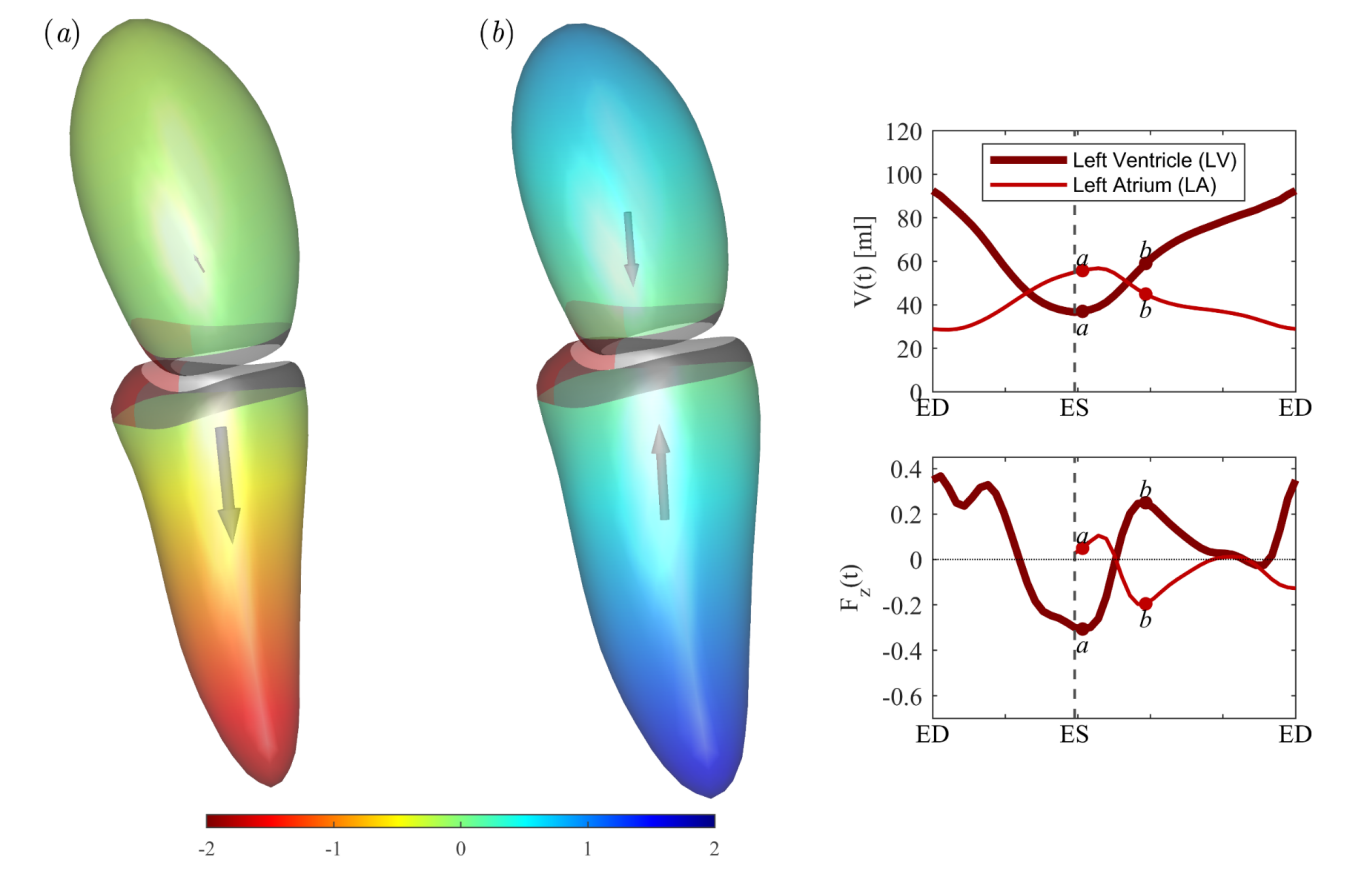
Diseased heart: Overview of the force at (**a**) the beginning of diastole, or suction phase, and (**b**) before the end of early filling, or inflow deceleration phase, for an average patient with hypertrophic cardiomyopathy. Pictures (**a,b**) display the geometry of left ventricle (LV) and left atrium (LA), colored by the relative pressure assumed zero at the LA-LV boundary, and the dimensionless force vector. The graphs on the right side report the time course of the volume of LA and LV (above) and the dimensionless force (below), with indication of the timing of pictures (**a,b**).

The results obtained in the patient population, reported on the right column of Fig. 1, present remarkable differences particularly during the early part of diastole. Tabular quantitative results are also reported in Table 2. The LV principal strain is essentially preserved with only a small increase in patients that matches with the small increase of $\:\text{E}\text{F}$. Differently, LA principal strain value reduces significantly; the reduction in the maximum value appears imputable to the modified behavior of passive contraction in the first filling wave of diastole (early wave, or E wave) as indicated by the drastically lowered strain rate. This is a generalized phenomenon that is noticeable in any component of strain tensor (reported in Supplemental Fig. S1): LV strain presents small changes that can be positive or negative depending on the strain component, whereas LA strain is generally reduced, and all strain curves feature a decrease of the slope during the first diastolic wave. The change in the relative LV-LA deformation, especially the reduction of strain during the E wave, testifies a reduction of LA elastic recovery, or an increase of LV stiffness, during the transit of blood from LA to LV in early diastole. This observation describes apparently minor changes in LV function that are accompanied by more evident alteration of LA function. The analysis of the forces exchanged between the two chambers shows that this behavior corresponds to a drastic modification of the LV-LA dynamic interaction and allows to better identify the specific phases when the pathological function differs from normal. At the end of systole and the onset of diastole, the LA force is close to zero, acceleration of flow from LA to LV is due to LV suction force due to active relaxation at the end of systole and this does not show any difference in the patient group (in agreement to the preservation of systolic function). Differently, immediately after this, during the early (passive) phase of diastole, the patients’ LA features a drastic reduction of the force pushing blood toward the LV and receives a comparable backward force from the rigid LV; as a consequence, the atrium can barely overcome the LV elastic resistance. A comparative perception of the difference between normal and diseased conditions in the early phase of diastole can be gained by Figs. 3 and 4, respectively.

**Table 2:** Relevant deformation and force parameters for the left ventricle (LV) and the left atrium (LA): principal strain (PS) at end systole, maximum principal strain rate (PSR), and maximum force during diastole.

	Normal (*n* = 50)	HCM (*n* = 81)	*p*
LV end-systolic GPS (%)	− 0.33 ± 0.03	− 0.38 ± 0.05	< 10^−6^
LA end-systolic GPS (%)	+ 0.61 ± 0.10	+ 0.47 ± 0.12	< 10^−6^
LV peak diastolic GPSR ($\:{T}^{-1}$)	+ 1.97 ± 0.37	+ 2.02 ± 0.62	0.06
LA peak diastolic GPSR ($\:{T}^{-1}$)	− 2.42 ± 044	− 1.76 ± 0.52	< 10^−6^
LV peak diastolic force (%)	+ 0.38 ± 0.18	+ 0.32 ± 0.15	0.10
LA peak diastolic force (%)	− 0.96 ± 0.45	− 0.47 ± 0.32	< 10^−6^

Values are reported as average ± standard deviation, for the normal subjects (normal) and for the patients (HCM), last column reports the p-value of a t-student test for the hypothesis that the two groups are independent samples with equal mean.

## Discussion

Patients with HCM are often subjected to a progression toward the clinical syndrome of HF with symptoms and exercise intolerance, despite the normal systolic contraction testified by a preserved $\:\text{E}\text{F}$. However, they often show a small reduction of the end-systolic longitudinal strain^21^; therefore, the biomechanical roots of this pathology and, in particular, of its progression, are unclear. Understanding its origin can be of paramount relevance for the early diagnosis and the treatment of HFpEF, a clinical syndrome with a growing social impact^22^.

The present simultaneous analysis of LV and LA, in a selected population, allowed to recognize that the small alterations of LV function are associated with a significant reduction of LA strain, testifying the presence of a LA dysfunction. A reduction of LA strain and LA dysfunction are commonly encountered in HCM patient, of all ages, and it is associated with poor prognosis and HF^20,23,24^.

Strain is an integral and periodic curve: LV strain starts from zero at ED, decreases to its minimum during systolic contraction and increases during diastolic expansion returning to zero at ED; similarly, LA strain increases to its maximum during systolic stretch and returns to zero after diastole. Therefore, an alteration of the peak strain can be imputable, in principle, to either the systolic or the diastolic phase. The analysis of the strain curves demonstrate that the reduction of LA strain is imputable to the reduction of LA elastic recovery during the early phase of diastole, which in turn is imputable to the increased LV stiffness. This is testified by the reduction of the strain rate in that period, which is noticeable to a lesser extend in the LV as well, and in all components of strain. Results support the hypothesis that this pathology is primarily associated with a purely diastolic mechanical alteration, in the early (passive) phase of diastole, when both LA and LV chamber are connected and the modified structure on the LV side induces a dysfunction that is more evident on the other, weaker chamber.

The analysis in terms of deformations is mainly descriptive; in dynamic terms, it corresponds to an alteration in the forces exchanged between LA and LV during diastole, where the increased LV stiffness inhibits the transit of blood from the LA during passive filling. These observations suggest that, during diastole, the LA tissues are subjected to an abnormal stress condition following a dynamic unbalance between LV and LA that may pave the road to a progression of the dysfunction. The LA, as the weaker chamber, is forced in a sustained stretched condition that induces its progressive suffering, with progressive dilatation and reduction of function.

This is a purely diastolic phenomenon, occurring when the mitral valve is open and the LA-LV are connected virtually forming a single chamber and the alteration on one side (LV stiffening) reflects on the other side (LA lack of elastic recovery, sustained stretched condition and dilatation). A spiral of events that can progress toward HF due to failure of both LA emptying and LV filling, despite the good LV pumping systolic function.

The same approach can be translated with minor adaptations to the right side of the heart, where flow-related forces can have an even higher relevance due to the lower pressure of pulmonary circulation, to purse a comprehensive assessment of the total heart function.

This interdisciplinary study identifies the physical basis of diastolic HF, lying on the dynamic interaction between LV and left atrium during the LV filling phase. Biochemical-biomechanical mechanisms that lay at the basis of cardiac adaptation are extremely complex, and extension to the LA reserves further difficulties. These observational results may provide a firm physics-based ground for both physics/engineering modeling of heart function and to cardiologists for the design of clinical studies.

It is important to remark, however, that the present results were obtained by studying a specific group of young subjects. On the one hand this choice reduces the presence of concomitant pathologies and ensures high quality echocardiography; on the other, it represents a clinical limitation: children are in a developmental phase, a condition that may affect cardiac progressive adaptation, and additional studies are required before results can be generalized to different, adult populations.

## Materials and methods

### Clinical part

The analysis included a group of 81 children with hypertrophic cardiomyopathy (HCM) and 50 age-matched normal volunteers. Young patients ensured absence of confounding factors due to co-morbidities; moreover, young subjects allow recording of high-quality ultrasound images. The study was approved by the Institutional Review Board of the Children’s Hospital of Philadelphia, USA and was carried out in accordance with the principles of the Declaration of Helsinki. All subjects or their guardians provided informed consent to participate to this research.

HCM was defined as per the American College of Cardiology and the American Heart Association guidelines for unexplained left ventricular hypertrophy in the absence of another cardiac or systemic disease^25^. In our study, HCM was defined as maximal LV septal or posterior wall thickness exceeding Boston pediatric z scores of + 2 at end-diastole.

**Fig. 5:**
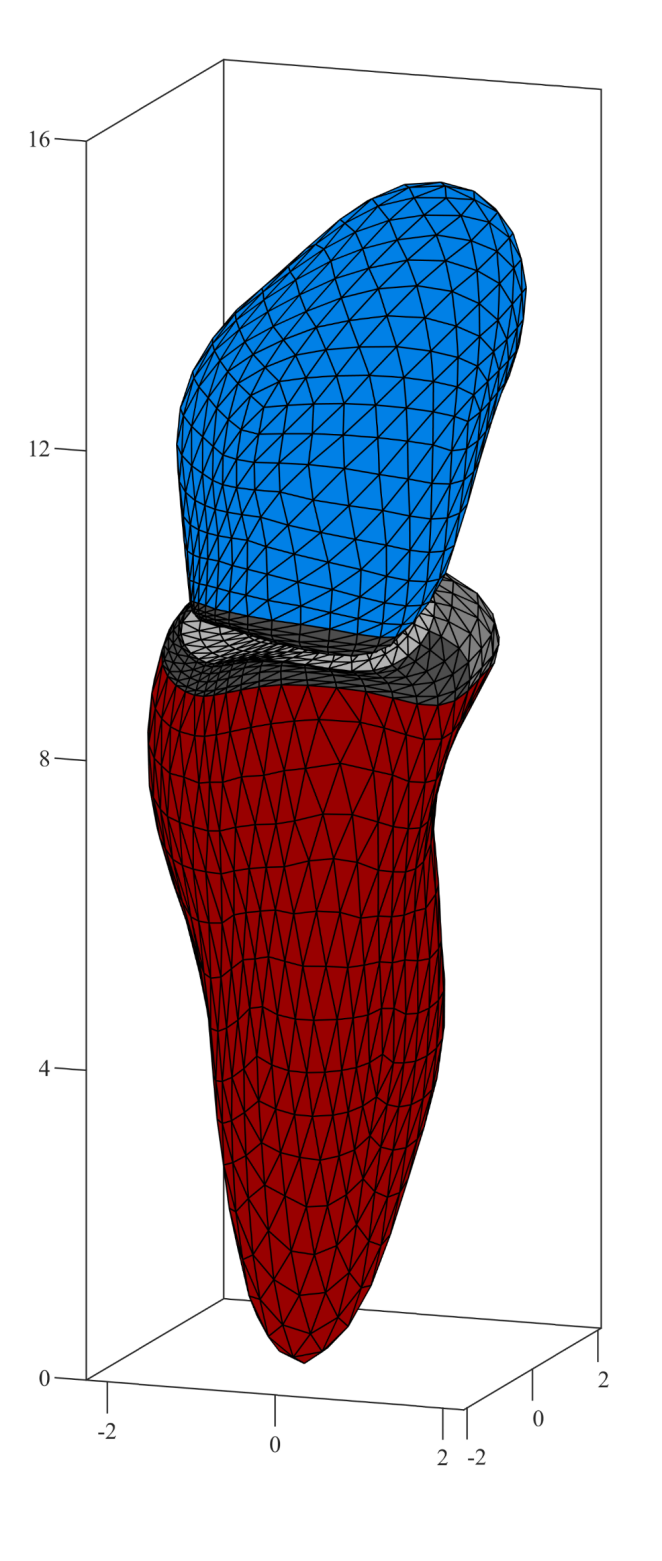
Example of a triangulated geometry extracted from the 3D echocardiography. Left ventricle is colored in red, left atrium in blue, the valvular regions are reported in shadows of gray.

All subjects underwent a three-dimensional (3D) Transthoracic Echocardiography (TTE) conducted with a Philips ultrasound system (EPIQ CV9; Philips Healthcare, Andover, Massachusetts) and a 3D matrix probe (X5-1c). TTE was optimized to record both LV and LA on the same scan to ensure that the analyses of the two chambers were perfectly synchronized and belong to the same heartbeat. LV and LA were then were analyzed by the clinical software 4D LV Analysis (TomTec Imaging Systems GmbH, Unterschleissheim, Germany) that performs a 3D tissue tracking by optical flow methods and returns the surface $\:S\left(t\right)$ of the internal boundary of the chamber in triangulated form, where the coordinated of the vertices identify material points moving during the heartbeat. The software is designed for the LV, allowing the user to control the LV boundaries and the location of the mitral and aortic valves. It was applied here for LA analysis, taking care to ensure a common position of the mitral valve and orientation, and provide, for the first time, a unitary 3D analysis for the function of the two chambers. To this purpose, LA was obtained starting from the same analysis image used to obtain LV and, keeping the MV section blocked, the apex is rotated 180° from LV towards LA; then, a second 180° rotation is performed clockwise around the axis in order to have the correct orientation of the aortic position, used as a reference point. Additionally, to minimize anatomical effect, we carefully traced the LA endocardial surface, excluding the LA appendage and pulmonary veins; afterwards, the processing is done as for LV. As both LV and LA were evaluated from the same scan, the extracted geometries had common coordinates and automatically placed in correct relative position; one illustrative example is reported in Fig. 5.

### Physics-based evaluations

The triangulated boundaries were exported and analyzed with MatLab (MathWorks, Natick, Massachusetts, USA). First the volumes of the LV and LA chambers are evaluated from the moving boundary using the Gauss formula

2 $$\:\begin{array}{c}V\left(t\right)=\underset{S\left(t\right)}{\overset{}{\int\:}}\varvec{x}\cdot\:\varvec{n}dS,\end{array}$$

where the integral is evaluated by summation over all triangles, $\:\varvec{x}\left(t\right)$ is the position vector of the center, $\:\varvec{n}\left(\varvec{x},t\right)$ is the outward unit normal vector and $\:dS$ is the area. Secondly, the availability of the three-dimensional geometry allows the evaluation of the complete deformation tensor at every triangle describing the chamber surface (excluding the valvular regions) relative to the initial (ideally undeformed) condition commonly assumed as the end of LV diastole. The Lagrangian (or Biot or engineering) 2$\:\times\:$2 symmetric strain tensor is evaluated on every triangle by a standard procedure that was previously described in detail^26^; once the strain tensor is evaluated the eigenvectors are the principal strains and the first one, the most negative LV principal strain and the most positive LA principal strain, is of primary interest. The time course of its spatial average, $\:St\left(t\right)$, is reported as a global measure of LV and LA function. The first principal strain is used here because it represents the most comprehensive measure of the amount of deformation, while the second principal strain is much weaker^27,28^. Clinical studies report commonly the longitudinal and circumferential components of strain, that can be also computed from two-dimensional imaging; however, we preferred to use the principal strain in this study because the relative relevance between circumferential and longitudinal components may be influenced by specific details of a pathology. Nevertheless, it can be anticipated that the conclusions drawn from the results of this study do not vary significantly when using any component of the strain tensor.

The natural strain rate is then evaluated from the time derivative

3 $$\:\begin{array}{c}SR\left(t\right)=\frac{d}{dt}\text{log}\left(St\left(t\right)+1\right),\end{array}$$

and the logarithm is required to pass from a Lagrangian definition of strain to a natural definition of strain rate, a measure of instantaneous function independent from the starting undeformed condition. In all calculations, the time is normalized to the heartbeat duration, $\:T$; therefore the strain rate (3) takes unit equal to $\:{T}^{-1}$.

Chambers’ volume and deformation provide a description of the state and activity of the cardiac tissues; therefore, when alterations are detected, they reflect changes that have already occurred to the tissues. On the other hand, volumes and deformation do not tell about active actions, e.g. forces, that have been responsible for such alteration or may induce further progression.

The dynamic interaction between the tissue surrounding a chamber and the blood contained therein is evaluated by measuring the total force exchanged between them^14,29^. This force, $\:{\varvec{F}}^{*}\left(t\right)$, is given by the change of momentum inside each chamber, and can be evaluated by the flux of momentum across the triangulated surface, $\:S\left(t\right)$, surrounding the chamber^30^

4 $$\:\begin{array}{c}{\varvec{F}}^{*}\left(t\right)=\rho\:\underset{S\left(t\right)}{\overset{}{\int\:}}\left[\varvec{v}\left(\varvec{v}\cdot\:\varvec{n}\right)+\varvec{x}\left(\frac{\partial\:\varvec{v}}{\partial\:t}\cdot\:\varvec{n}\right)\right]dS,\end{array}$$

where $\:\rho\:$ is the blood density and $\:\varvec{v}\left(\varvec{x},t\right)$ is the velocity vector field. The integral is performed by summation of the contribution of each moving triangle taking care of properly handling the triangles pertaining to the tissue and describing flow section. On the solid part of the boundary, both tissue and closed walls, the velocity field is given by the velocity of the boundary itself, $\:v=\frac{\partial\:\varvec{x}}{\partial\:t}$. On flow sections, the open valve, the velocity is estimated as the average velocity due to mass conservation^31^

5 $$\:\begin{array}{c}\varvec{v}\left(x,t\right)=\frac{\partial\:\varvec{x}}{\partial\:t}-\frac{1}{A}\frac{\partial\:V}{\partial\:t}\varvec{n},\end{array}$$

where $\:A\left(t\right)$ is the flow area: the area of aorta during LV systole, and the area of mitral annulus, reduced by a standard contraction factor $\:\frac{\pi\:}{\pi\:+2}$, for both LV and LA calculations during LV diastole. The LA force is not computed during LV systole because the necessary information about pulmonary veins (position, size, orientation) is not available. The force (4) represents a volumetric balance and its dimensional value depends on the size of the chamber; therefore, it is normalized by the volume to avoid this variability; it is then divided by blood density and gravity acceleration, $\:g$, to have it expressed in dimensionless form as a fraction of gravity force

6 $$\:\begin{array}{c}\varvec{F}\left(t\right)=\frac{{\varvec{F}}^{*}\left(t\right)}{\rho\:gV\left(t\right)}.\end{array}$$

## Electronic supplementary material

Below is the link to the electronic supplementary material.

Supplementary Material 1

## Data Availability

The datasets used and analyzed during the current study available from the corresponding author on reasonable request.
