# Supplementary material for: A scenario for heart failure during the filling phase
Source: Sci Rep. 2024 Oct 1;14:22760. doi: 10.1038/s41598-024-74155-4 (PMC11445582; doi:10.1038/s41598-024-74155-4)
Supplement: Supplementary file 1 — Supplementary Material 1 [file 41598_2024_74155_MOESM1_ESM.pdf]

SUPPLEMENTARY MATERIAL

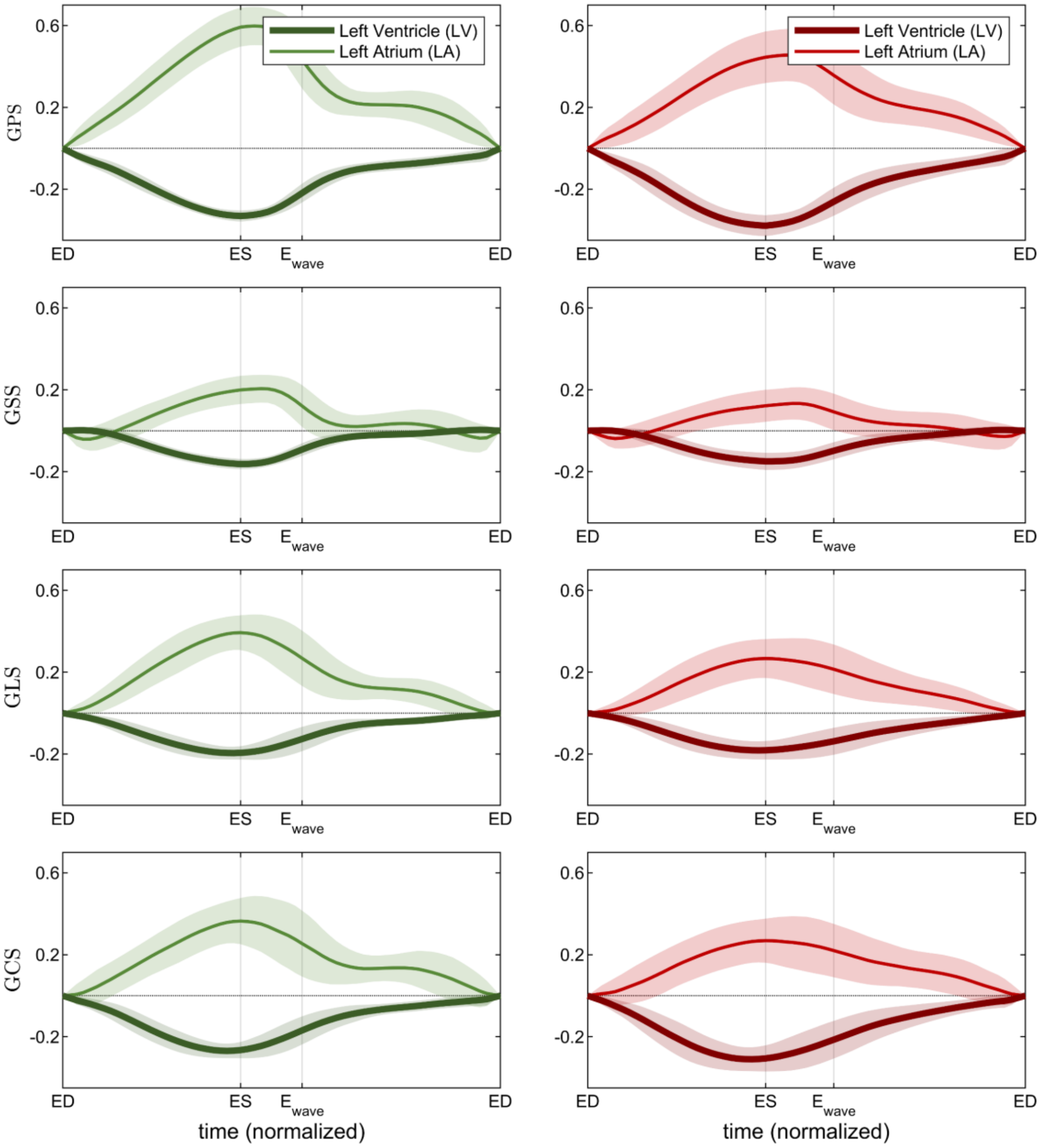

Supplemental Figure S.1. Time course of the different components of strain, in the left ventricle (thick line) and in the left atrium (light line), the line reports the average value and the surrounding shadow is ± the standard deviation. From top to bottom: global principal strain (GPS), the average of the highest strain (in absolute value); global secondary strain (GSS), the second principal strain; global longitudinal strain (GLS), strain in the base-apex direction; global circumferential strain (GCS), strain in the circumferential direction. Left column is evaluated from the normal population (50 subjects), right column from the matched patient with hypertrophic cardiomyopathy (81 subjects). Timing indicators are: ED=end diastole, ES=end systole, E_{wave}=early filling wave.
